# Supplementary figures and images for: Comparative Analysis of Urso- and Tauroursodeoxycholic Acid Neuroprotective Effects on Retinal Degeneration Models
Source: Pharmaceuticals (Basel). 2022 Mar 9;15(3):334. doi: 10.3390/ph15030334 (PMC8955596; doi:10.3390/ph15030334)

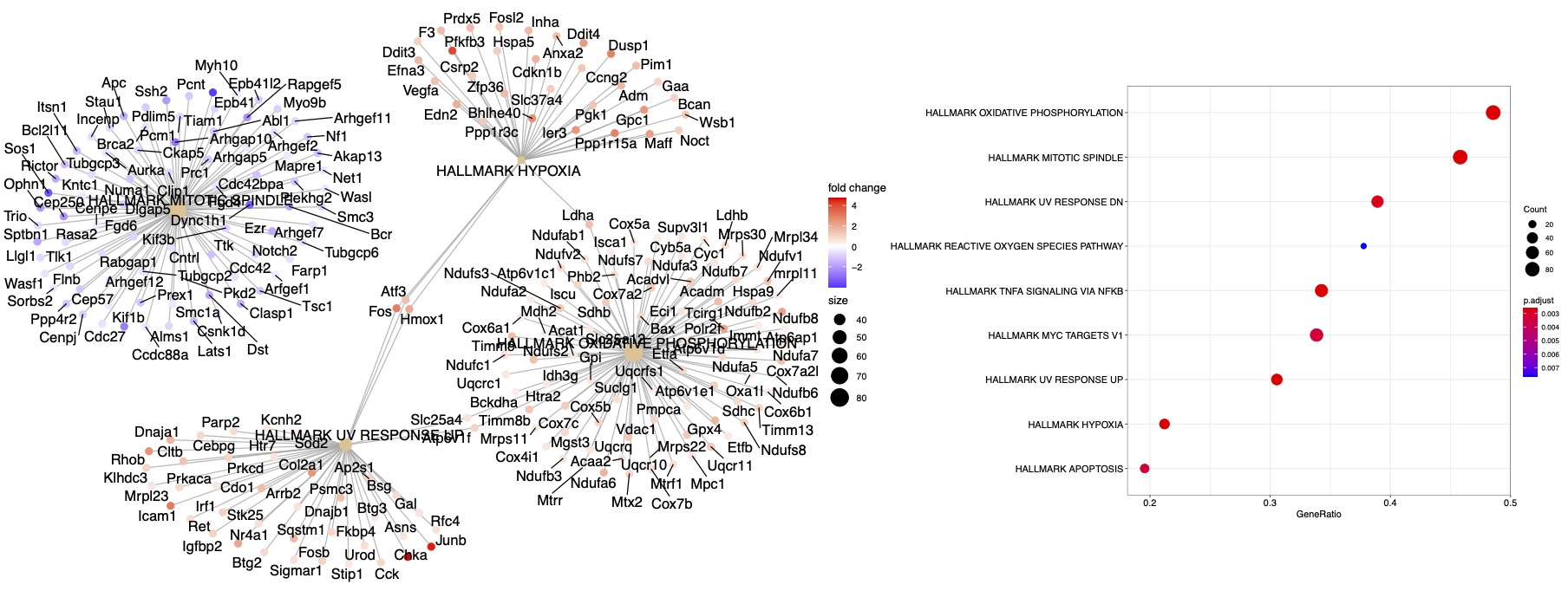

Supplement: Supplementary file 1 [file pharmaceuticals-15-00334-s001.zip › Supplementary figure1.jpg]
